# Hemolymphoreticular Neoplasias from the Ramazzini Institute Long-term Mice and Rat Studies on Aspartame

**DOI:** 10.5334/aogh.4163

**Published:** 2023-06-20

**Authors:** Federica Gnudi, Simona Panzacchi, Eva Tibaldi, Martina Iuliani, Daria Sgargi, Luciano Bua, Daniele Mandrioli

**Affiliations:** 1 Cesare Maltoni Cancer Research Center, Ramazzini Institute, Via Saliceto 3, 40010 Bentivoglio, IT

**Keywords:** aspartame, cancer, leukemia, lymphoma, bioassays

## Abstract

**Background:**

Haemolymphoreticular neoplasias (HLRNs) from the Ramazzini Institute (RI) carcinogenicity studies on Aspartame (APM) in rats and mice were heterogeneously grouped over the years and different statistical methods were applied.

**Objective:**

We report all the detailed HLRN diagnoses of all the RI rats and mice studies on APM and the related statistics.

**Methods:**

Histological subtypes and lineage (myeloid or lymphoid) are reported in males (MM) and females (FF) in line with the International Harmonization of Nomenclature and Diagnostic Criteria for Lesions (INHAND) for rodents and the World Health Organization (WHO) Classification of Tumours of Haematopoietic and Lymphoid Tissues. Statistical analyses included Fisher’s Exact test and Cochran-Armitage trend test.

**Findings:**

Results from the post-natal bioassay on Sprague-Dawley (SD) rats (BT6008) showed statistically significant increases in lymphomas (all types) (MM, FF), leukemias (all types) (FF), immunoblastic lymphomas (MM, FF), total lymphoid tumours (MM, FF), monocytic leukemia (FF), myeloid leukemia (FF), histiocytic sarcoma (FF), and total myeloid tumours (FF). Results from the prenatal experiment on SD rats (BT6009), showed statistically significant increases in lymphomas (all types) (FF), leukemias (all types) (FF), total lymphoid tumours (FF), myeloid leukemia (FF), and total myeloid tumours (FF). Finally, results from the prenatal bioassay on Swiss mice (BT6010) showed statistically significant increases in leukemias (all types) (MM, FF), lymphoblastic leukemia (MM, FF), monocytic leukemia (MM) and total myeloid tumours (MM).

**Conclusions:**

Our analyses, performed in line with international recommended guidelines for statistics and pathology, confirm and reinforce our previous findings of statistically significant increases of HLRNs in rodents exposed to APM.

## Introduction

In the 1990s, due to the extensive use of Aspartame (APM) in many products, the Ramazzini Institute (RI) started a series of carcinogenicity bioassays in order to test the effects of APM administration on rats and mice [1,2,3]. A total of 2,270 Sprague-Dawley (SD) rats and 852 Swiss mice were included in the studies with the aim to test the effects of both prenatal and post-natal APM long-term exposure [1,2,3,4]. Results supported the hypothesis that APM is a multisite experimental carcinogen in rodents inducing significant increased incidences of several types of malignant tumours [5]. However, haemolymphoreticular neoplasias (HLRNs) from the RI carcinogenicity studies on APM in rats and mice were heterogeneously grouped over the years and different statistical methods were applied. In general, HLRNs were previously reported in all RI studies as a single group, without detailed reporting of the tumour subtypes [i.e. monocytic leukemia, myeloid leukemia, etc.]. In particular, the postnatal study on SD rats (BT6008) showed an increased trend of total HLRNs in male and female rats following APM exposure and statistically significant increases in groups treated with doses equal or higher than 400 ppm in female rats [1]. Moreover, results from the prenatal study on SD rats (BT6009) showed significant increased trend of HLRNs and a statistically significant increase in the group treated with the highest dose (2,000 ppm) in female rats [2]. In the prenatal study on mice (BT6010), no statistically significant increase of HLRNs was reported [3]. In 2020, HLRNs diagnosed in the BT6009 experiment were re-evaluated by the RI also with the use of immunohistochemistry and the statistical analysis was performed on both the total incidences of HLRNs within each treatment group, as well as on the individual incidences by histotype [4]. In female rats, the statistical analysis confirmed the statistically significant increase in total HLRNs and showed a statistically significant positive trend in lymphomas (all types), leukemias (all types) in the treated groups and a statistically significant increased incidence in the group treated with the highest dose (2,000 ppm). Finally, a statistically significant positive trend of myeloid leukemia was also observed in female rats.

Here we report all the detailed HLRNs original diagnoses of all the rats and mice studies performed by the RI, including histological subtypes and lineage (myeloid or lymphoid) in line with International Harmonization of Nomenclature and Diagnostic Criteria for Lesions (INHAND) and the World Health Organization (WHO) Classification of Tumours of Haematopoietic and Lymphoid Tissues [6,7,8,9]. The hematopoietic system derives from a multipotent primitive cell that can develop into all types of blood cells, including myeloid-lineage and lymphoid-lineage cells. Thus, the basic principle of these classifications is to report diseases by subtype and by lineage (myeloid and lymphoid) [7,8,9]. A statistical re-evaluation was performed using Fisher’s Exact test and Cochran-Armitage trend test. These statistical methods have been already applied in the more recent publications of the RI and are in compliance with the OECD and IARC recommendations for analyzing and interpreting life-span carcinogenicity studies [10,11,12,13,14].

## Materials and Methods

### RI long-term carcinogenicity bioassays on APM: study design (BT6008, BT6009, BT6010)

Pathological data including original diagnoses from long-term carcinogenicity bioassays on APM (BT6008, BT6009, BT6010) [1,3,4] were retrieved from previous publications and the RI archive. For BT6009 we included the pathological diagnoses after re-evaluation by the RI with the use of immunohistochemistry [4]. In BT6008, APM was administered with feed *ad libitum* to male and female Sprague-Dawley (SD) rats (100–150/sex/group) of eight weeks of age until natural death. The concentrations tested were: 100,000, 50,000, 10,000, 2,000, 400, 80 or 0 ppm [1]. In BT6009, APM was administered with feed *ad libitum* to male and female SD rats (70–95/sex/group) from prenatal life (12^th^ day of pregnancy) until natural death. The concentrations tested were: 2,000, 400 or 0 ppm [2,4]. Finally, in BT6010, APM was administered with feed *ad libitum* to male and female Swiss mice (62–122/sex/group) from prenatal life (12^th^ day of pregnancy) until natural death. The concentrations tested were: 32,000, 16,000, 8,000, 2,000or 0 ppm [3] In all the experiments, control animals received the same feed without APM. For each experiment, the pathological data are reported in tables including total HLRNs, leukemias (all types), lymphomas (all types), histiocytic sarcoma, histological subtypes, total myeloid tumours, total lymphoid tumours. Pathological data are reported in line with the INHAND and the WHO Classification of Tumours of Haematopoietic and Lymphoid Tissues [6,7,8,9]. The present study did not require the use of animals, therefore no ethics approval was needed. Ethics statement related to the animal studies BT6008, BT6009, BT6010 are reported in the original manuscripts [1,2,3].

#### Statistical analysis

Statistical methods are in compliance with the IARC recommendations and the OECD international guidelines for life-span carcinogenicity studies [12,13,14]. Differences between each treatment group and their respective control were analysed separately using the Fisher Exact test (reported as one or two-tailed). Furthermore, the Cochran-Armitage trend test was applied to evaluate the statistical significance of linear trends according to the dose administered. The significance level adopted for all analyses was 0.05. All statistical elaborations were performed using Stata 17 (StataCorp LLC). Previously published statistically significant results were also reported in the tables.

## Results

### Long-term carcinogenicity bioassays on APM administered with feed to Sprague-Dawley rats from eight weeks of age (Experiment BT6008) (Tables 1–2)

**Table 1:** Long-term carcinogenicity bioassay on APM administered *ad libitum* with feed to Sprague-Dawley rats from 8 weeks of age (BT6008): animals bearing HLRN, lymphoma (all types), histiocytic sarcoma, leukemia (all types).


GROUP	DOSE PPM (mg/kg bw)	ANIMALS		ANIMALS BEARING HLRN^a^				LYMPHOMAS (ALL TYPES)^a^			HISTIOCYTIC SARCOMA^a^			LEUKEMIAS (ALL TYPES)^a^		
				
		SEX	NO.	NO.	%			NO.	%		NO.	%		NO.	%	

I	100000	M	100	29	29.0			24	24.0	*P = 0.016; **P = 0.026	3	3.0		2	2.0	

	(5000)	F	100	25	25.0	○○	*P < 0.001; **P = 0.001	14	14.0	*P = 0.028; **P = 0.043	7	7.0		4	4.0	*P = 0.025; **P = 0.025

II	50000	M	100	20	20.0			13	13.0		6	6.0		1	1.0	

	(2500)	F	100	25	25.0	○○	*P < 0.001; **P = 0.001	12	12.0		8	8.0	*P = 0.053	5	5.0	*P = 0.010; **P = 0.010

III	10000	M	100	15	15.0			8	8.0		3	3.0		4	4.0	

	(500)	F	100	19	19.0	○	*P = 0.015; **P = 0.020	7	7.0		10	10.0	*P = 0.015; **P = 0.022	2	2.0	

IV	2000	M	150	33	22.0			16	10.7		10	6.7		7	4.7	

	(100)	F	150	28	18.7	○	*P = 0.009; **P = 0.018	14	9.3		8	5.3		6	4.0	*P = 0.015; **P = 0.030

V	400	M	150	25	16.7			22	14.7		2	1.3		1	0.7	

	(20)	F	150	30	20.0	○○	*P = 0.004; **P = 0.008	17	11.3		9	6.0		5	3.3	*P = 0.030

VI	80	M	150	23	15.3			15	10.0		4	2.7		4	2.7	

	(4)	F	150	22	14.7			14	9.3		6	4.0		2	1.3	

VII	0	M	150	31	20.7	◊ ○	**#P = 0.0173**	19	12.7	**#P = 0.0016**	8	5.3		4	2.7	

	0	F	150	13	8.7	◊◊ ○	**#P = 0.0048**	9	6.0		4	2.7		0	0.0	


^a^ Percentages refer to the number of animals at start. Previous statistical analyses (Soffritti et al., 2006). ◊ Statistically significant (p ≤ 0.05) using Cochran-Armitage test. ◊◊ Statistically significant (p ≤ 0.01) using Cochran-Armitage test. ○ Statistically significant (p ≤ 0.05) using poly-k test (k = 3). ○○ Statistically significant (p ≤ 0.01) using poly-k test (k = 3). **New statistical analyses (in bold):** * One-tailed Fisher’s exact test; ** Two-tailed Fisher’s exact test. **# Cochran–Armitage test for trend**.

**Table 2:** Long-term carcinogenicity bioassay on APM administered *ad libitum* with feed to Sprague-Dawley rats from 8 weeks of age (BT6008): lymphoid and myeloid tumours.


GRO-UP	DOSE PPM (mg/kg bw)	ANIMALS		ANIMALS BEARING HLRN A																							

				LYMPHO-BLASTIC LYM-PHOMA		LYMPHO-BLASTIC LEUKE-MIA		LYMPHO-CYTIC LYM-PHOMA		IMMUNO-BLASTIC LYM-PHOMA			TOTAL LYMPH-OID TUMORS			MONO-CYTIC LEUKE-MIA			MYEL-OID LEUKE-MIA			HISTIO-CYTIC SAR-COMA			TOTAL MYEL-OID TUMORS		
									
		SEX	NO.	NO.	%	NO.	%	NO.	%	NO.	%		NO.	%		NO.	%		NO.	%		NO.	%		NO.	%	

I	100000	M	100	0	0.0	0	0.0	0	0.0	24	24.0	*P = 0.016; **P = 0.026	24	24.0	*P = 0.023; **P = 0.041	1	1.0		1	1.0		3	3.0		5	5.0	

	(5000)	F	100	1	1.0	0	0.0	2	2.0	11	11.0	*P = 0.016; **P = 0.019	14	14.0	*P = 0.028; **P = 0.043	2	2.0		2	2.0		7	7.0		11	11.0	*P = 0.008; **P = 0.012

II	50000	M	100	0	0.0	0	0.0	0	0.0	13	13.0		13	13.0		1	1.0		0	0.0		6	6.0		7	7.0	

	(2500)	F	100	2	2.0	0	0.0	0	0.0	10	10.0	*P = 0.030; **P = 0.053	12	12.0		4	4.0	*P = 0.025; **P = 0.025	1	1.0		8	8.0	*P = 0.053	13	13.0	*P = 0.002; **P = 0.003

III	10000	M	100	0	0.0	1	1.0	0	0.0	8	8.0		9	9.0		2	2.0		1	1.0		3	3.0		6	6.0	

	(500)	F	100	2	2.0	0	0.0	2	2.0	3	3.0		7	7.0		2	2.0		0	0.0		10	10.0	*P = 0.015; **P = 0.022	12	12.0	*P = 0.004; **P = 0.006

IV	2000	M	150	0	0.0	0	0.0	1	0.7	15	10.0		16	10.7		7	4.7		0	0.0		10	6.7		17	11.3	

	(100)	F	150	5	3.3	1	0.7	1	0.7	8	5.3		15	10.0		4	2.7		1	0.7		8	5.3		13	8.7	*P = 0.021; **P = 0.043

V	400	M	150	0	0.0	0	0.0	0	0.0	22	14.7		22	14.7		1	0.7		0	0.0		2	1.3		3	2.0	

	(20)	F	150	7	4.7	0	0.0	2	1.3	8	5.3		17	11.3		5	3.3	*P = 0.030	0	0.0		9	6.0		14	9.3	*P = 0.013; **P = 0.026

VI	80	M	150	3	2.0	0	0.0	0	0.0	12	8.0		15	10.0		2	1.3		2	1.3		4	2.7		8	5.3	

	(4)	F	150	3	2.0	0	0.0	5	3.3	6	4.0		14	9.3		2	1.3		0	0.0		6	4.0		8	5.3	

VII	0	M	150	0	0.0	1	0.7	0	0.0	19	12.7	**#P = 0.0006**	20	13.3	**#P = 0.0022**	2	1.3		1	0.7		8	5.3		11	7.3	

	(0)	F	150	2	1.3	0	0.0	2	1.3	5	3.3	**#P = 0.0015**	9	6.0		0	0.0		0	0.0	**#P = 0.0060**	4	2.7		4	2.7	**#P = 0.0473**


^a^ Percentages refer to the number of animals at start. **New statistical analyses (in bold):** * One-tailed Fisher’s exact test ; ** Two-tailed Fisher’s exact test. **# Cochran–Armitage test for trend**.

The following statistically significant increased trends were observed among treated groups: animals bearing HLRN (MM: P = 0.0173 Cochran-Armitage trend test; FF: P = 0.0048 Cochran-Armitage trend test), lymphoma (all types) (MM: P = 0.0016 Cochran-Armitage trend test), immunoblastic lymphoma (MM: P = 0.0006 Cochran-Armitage trend test; FF: P = 0.0015 Cochran-Armitage trend test), total lymphoid tumours (MM: P = 0.0022 Cochran-Armitage trend test), myeloid leukemia (FF: P = 0.0060 Cochran-Armitage trend test) and total myeloid tumours (FF: P = 0.0473 Cochran-Armitage trend test).

The following statistically significant increased incidences were observed in treated groups: animals bearing HLRN (FF: 100,000 ppm, P < 0.001 one-tailed Fisher’s Exact test, P = 0.001 two-tailed Fisher’s Exact test; 50,000 ppm, P < 0.001 one-tailed Fisher’s Exact test, P = 0.001 two-tailed Fisher’s Exact test; 10,000 ppm, P = 0.015 one-tailed Fisher’s Exact test, P = 0.020 two-tailed Fisher’s Exact test; 2,000 ppm, P = 0.009 one-tailed Fisher’s Exact test, P = 0.018 two-tailed Fisher’s Exact test; 400 ppm, P = 0.004 one-tailed Fisher’s Exact test, P = 0.008 two-tailed Fisher’s Exact test); lymphomas (all types) (MM: 100,000 ppm, P = 0.016 one-tailed Fisher’s Exact test, P = 0.026 two-tailed Fisher’s Exact test; FF: 100,000 ppm, P = 0.028 one-tailed Fisher’s Exact test, P = 0.043 two-tailed Fisher’s Exact test), leukemias (all types) (FF: 100,000 ppm, P = 0.025 one-tailed Fisher’s Exact test, P = 0.025 two-tailed Fisher’s Exact test; 50,000 ppm, P = 0.010 one-tailed Fisher’s Exact test, P = 0.010 two-tailed Fisher’s Exact test; 2,000 ppm, P = 0.015 one-tailed Fisher’s Exact test, P = 0.030 two-tailed Fisher’s Exact test; 400 ppm, P = 0.030 one-tailed Fisher’s Exact test), immunoblastic lymphoma (MM: 100,000 ppm, P = 0.016 one-tailed Fisher’s Exact test, P = 0.026 two-tailed Fisher’s Exact test; FF: 100,000 ppm, P = 0.016 one-tailed Fisher’s Exact test, P = 0.019 two-tailed Fisher’s Exact test; 50,000 ppm, P = 0.030 one-tailed Fisher’s Exact test, P = 0.053 two-tailed Fisher’s Exact test), total lymphoid tumours (MM: 100,000 ppm, P = 0.023 one-tailed Fisher’s Exact test, P = 0.041 two-tailed Fisher’s Exact test; FF: 100,000 ppm, P = 0.028 one-tailed Fisher’s Exact test, P = 0.043 two-tailed Fisher’s Exact test), monocytic leukemia (FF: 50,000 ppm, P = 0.025 one-tailed Fisher’s Exact test, P = 0.025 two-tailed Fisher’s Exact test; 400 ppm, P = 0.030 one-tailed Fisher’s Exact test), histiocytic sarcoma (FF: 50,000 ppm, P = 0.053 one-tailed Fisher’s Exact test; 10,000 ppm, P = 0.015 one-tailed Fisher’s Exact test, P = 0.022 two-tailed Fisher’s Exact test) and total myeloid tumours (FF: 100,000 ppm, P = 0.008 one-tailed Fisher’s Exact test, P = 0.012 two-tailed Fisher’s Exact test; 50,000 ppm, P = 0.002 one-tailed Fisher’s Exact test, P = 0.003 two-tailed Fisher’s Exact test; 10,000 ppm, P = 0.004 one-tailed Fisher’s Exact test, P = 0.006 two-tailed Fisher’s Exact test; 2,000 ppm, P = 0.021 one-tailed Fisher’s Exact test, P = 0.043 two-tailed Fisher’s Exact test; 400 ppm, P = 0.013 one-tailed Fisher’s Exact test, P = 0.026 two-tailed Fisher’s Exact test).

### Long-term carcinogenicity bioassays on APM administered with feed to Sprague-Dawley rats from prenatal life (Experiment BT6009) (Tables 3–4)

**Table 3:** Long-term carcinogenicity bioassay on APM administered *ad libitum* with feed to Sprague-Dawley rats from prenatal life (BT6009): animals bearing HLRN, lymphoma (all types), histiocytic sarcoma, leukemia (all types).


GROUP	DOSE PPM (mg/kg bw)	ANIMALS		ANIMALS BEARING HLRN^a^				LYMPHOMAS (ALL TYPES)^a^				HISTIOCYTIC SARCOMA^a^		LEUKEMIAS (ALL TYPES)^a^			
				
		SEX	NO.	NO.	%			NO.	%			NO.	%		NO.	%	

I	2000	M	70	10	14.3			9	12.9				0	0.0	1	1.4	

	(100)	F	70	21	30.0	••	*P = 0.005; **P = 0.010	16	22.9	•	*P = 0.027, **P = 0.050	1	1.4	4	5.7	•	*P = 0.031; **P = 0.031

II	400	M	70	9	12.9			6	8.6			1	1.4	2	2.9		

	(20)	F	70	12	17.1			11	15.7			0	0.0	1	1.4		

III	0	M	95	8	8.4			8	8.4			0	0.0	0	0.0		

	(0)	F	95	12	12.6	◊◊	**#P = 0.0045**	10	10.5	◊	**#P = 0.0368**	2	2.1	0	0.0	◊	**#P = 0.0106**


^a^ Percentages refer to the number of animals at start. Previous statistical analyses (Tibaldi et al., 2020): • Statistically significant (P ≤ 0.05) using Chi2 or Fisher exact test. •• Statistically significant (P ≤ 0.01) using Chi2 or Fisher exact test. ◊ Near the control incidence are the p-values (P ≤ 0.05) associated with the Cochran Armitage for the analysis of the trend. ◊◊ Near the control incidence are the p-values (P ≤ 0.01) associated with the Cochran Armitage for the analysis of the trend. **New statistical analyses (in bold):** * One-tailed Fisher’s exact test; ** Two-tailed Fisher’s exact test. **# Cochran–Armitage test for trend**.

**Table 4:** Long-term carcinogenicity bioassay on APM administered *ad libitum* with feed to Sprague-Dawley rats from prenatal life (BT6009): lymphoid and myeloid tumours.


GROUP	DOSE PPM (mg/kg bw)	ANIMALS		ANIMALS BEARING HLRN^a^																							

				LYMPHO-BLASTIC LYMPHOMA		LARGE GRANULAR LYMPHOCYTE LEUKEMIA		LYMPHO-CYTIC LYMPHOMA		IMMUNO-BLASTIC LYMPHOMA		PLASMA-CYTIC LYMPH-OMA		TOTAL LYMPH-OID TUMORS			MONO-CYTIC LEUK-EMIA		MYELOID LEUKEMIA				HISTIO-CYTIC SARCOMA		TOTAL MYELOID TUMORS		
										
		SEX	NO.	NO.	%	NO.	%	NO.	%	NO.	%	NO.	%	NO.	%		NO.	%	NO.	%			NO.	%	NO.	%	

I	2000	M	70	6	8.6	1	1.4	0	0.0	3	4.3	0	0.0	10	14.3		0	0.0	0	0.0			0	0.0	0	0.0	

	(100)	F	70	5	7.1	0	0.0	7	10.0	3	4.3	1	1.4	16	22.9	*P = 0.027; **P = 0.050	2	2.9	2	2.9			1	1.4	5	7.1	

II	400	M	70	5	7.1	0	0.0	0	0.0	1	1.4	0	0.0	6	8.6		2	2.9	0	0.0			1	1.4	3	4.3	

	(20)	F	70	5	7.1	0	0.0	3	4.3	3	4.3	0	0.0	11	15.7		1	1.4	0	0.0			0	0.0	1	1.4	

III	0	M	95	4	4.2	0	0.0	0	0.0	4	4.2	0	0.0	8	8.4		0	0.0	0	0.0			0	0.0	0	0.0	

	(0)	F	95	3	3.2	0	0.0	5	5.3	2	2.1	0	0.0	10	10.5	**#P = 0.0368**	0	0.0	0	0.0	◊	**#P = 0.0324**	2	2.1	2	2.1	**#P = 0.0485**


Previous statistical analyses (Tibaldi et al., 2020): ^a^ Percentages refer to the number of animals at start. • Statistically significant (P ≤ 0.05) using Chi2 or Fisher exact test. •• Statistically significant (P ≤ 0.01) using Chi2 or Fisher exact test. ◊ Near the control incidence are the p-values (P ≤ 0.05) associated with the Cochran Armitage for the analysis of the trend. ◊◊ Near the control incidence are the p-values (P ≤ 0.01) associated with the Cochran Armitage for the analysis of the trend. **New statistical analyses (in bold):** * One-tailed Fisher’s exact test ; ** Two-tailed Fisher’s exact test. **# Cochran–Armitage test for trend**.

The following statistically significant increased trends were observed among treated groups: animals bearing HLRN (FF: P = 0.0045 Cochran-Armitage trend test), lymphomas (all types) (FF: P = 0.0368 Cochran-Armitage trend test), leukemias (all types) (FF: P = 0.0106 Cochran-Armitage trend test), total lymphoid tumours (FF: P = 0.0368 Cochran-Armitage trend test), myeloid leukemia (FF: P = 0.0324 Cochran-Armitage trend test) and total myeloid tumours (FF: P = 0.0485 Cochran-Armitage trend test).

The following statistically significant increased incidences were observed in treated groups: animals bearing HLRN (FF: 2,000 ppm, P = 0.005 one-tailed Fisher’s Exact test, P = 0.010 two-tailed Fisher’s Exact test), lymphomas (all types) (FF: 2,000 ppm, P = 0.027 one-tailed Fisher’s Exact test, P = 0.050, two-tailed Fisher’s Exact test), leukemias (all types) (FF: 2,000 ppm, P = 0.031 one-tailed Fisher’s Exact test, P = 0.031 two-tailed Fisher’s Exact test) and total lymphoid tumours (FF: 2,000 ppm, P = 0.027 one-tailed Fisher’s Exact test, P = 0.050 two-tailed Fisher’s Exact test).

### Long-term carcinogenicity bioassays on APM administered with feed to Swiss mice from prenatal life (Experiment BT6010) (Tables 5–6)

**Table 5:** Long-term carcinogenicity bioassay on APM administered *ad libitum* with feed to Swiss mice from prenatal life (BT6010): animals bearing HLRN, lymphoma (all types), histiocytic sarcoma, leukemia (all types).


GROUP	DOSE PPM (mg/kg bw)	ANIMALS		ANIMALS BEARING HLRN^a^			LYMPHOMAS (ALL TYPES)^a^		HISTIOCYTIC SARCOMA^a^		LEUKEMIAS (ALL TYPES)^a^		
				
		SEX	NO.	NO.	%		NO.	%	NO.	%	NO.	%	

I	32000	M	83	12	14.5		3	3.6	0	0.0	9	10.8	*P = 0.007; **P = 0.009

	(4000)	F	62	25	40.3		12	19.4	2	3.2	11	17.7	

II	16000	M	64	12	18.8	*P = 0.040	2	3.1	0	0.0	10	15.6	*P = 0.001; **P = 0.001

	(2000)	F	64	16	25.0		6	9.4	2	3.1	8	12.5	

III	8000	M	62	10	16.1		1	1.6	0	0.0	9	14.5	*P = 0.001; **P = 0.001

	(1000)	F	73	24	32.9		9	12.3	1	1.4	14	19.2	

IV	2000	M	103	10	9.7		1	1.0	0	0.0	9	8.7	*P = 0.018; **P = 0.026

	(250)	F	122	54	44.3		16	13.1	4	3.3	34	27.9	*P = 0.007; **P = 0.014

V	0	M	117	10	8.5		7	6.0	1	0.9	2	1.7	**#P = 0.0492**

	(0)	F	102	39	38.2		18	17.6	7	6.9	14	13.7	


^a^ Percentages refer to the number of animals at start. HLRN data unpublished. **New statistical analyses (in bold):** * One-tailed Fisher’s exact test; ** Two-tailed Fisher’s exact test. **# Cochran–Armitage test for trend**.

**Table 6:** Long-term carcinogenicity bioassay on APM administered ad libitum with feed to Swiss mice from prenatal life (BT6010): lymphoid and myeloid tumours.


GROUP	DOSE PPM (mg/kg bw)	ANIMALS		ANIMALS BEARING HLRN^a^																				

				LYMPHO-BLASTIC LYMPHOMA		LYMPHO-BLASTIC LEUKEMIA			LYMPHO-CYTIC LYMPHOMA		IMMUNO-BLASTIC LYMPHOMA		TOTAL LYMPHOID TUMORS		MONO-CYTIC LEUKEMIA			MYELOID LEUKEMIA		HISTIO-CYTIC SARCOMA		TOTAL MYELOID TUMORS		
									
		SEX	NO.	NO.	%	NO.	%		NO.	%	NO.	%	NO.	%	NO.	%		NO.	%	NO.	%	NO.	%	

I	32000	M	83	2	2.4	6	7.2	*P = 0.021; **P = 0.021	1	1.2	0	0.0	9	10.8	0	0.0		3	3.6	0	0.0	3	3.6	
		
	(4000)	F	62	7	11.3	6	9.7		3	4.8	2	3.2	18	29.0	4	6.5		1	1.6	2	3.2	7	11.3	

II	16000	M	64	0	0.0	4	6.3	*P = 0.054; **P = 0.054	0	0.0	2	3.1	6	9.4	3	4.7	*P = 0.043; **P = 0.043	3	4.7	0	0.0	6	9.4	*P = 0.024; **P = 0.024
		
	(2000)	F	64	5	7.8	7	10.9		0	0.0	1	1.6	13	20.3	1	1.6		0	0.0	2	3.1	3	4.7	

III	8000	M	62	1	1.6	8	12.9	*P = 0.001; **P = 0.001	0	0.0	0	0.0	9	14.5	1	1.6		0	0.0	0	0.0	1	1.6	
		
	(1000)	F	73	6	8.2	12	16.4		1	1.4	2	2.7	21	28.8	2	2.7		0	0.0	1	1.4	3	4.1	

IV	2000	M	103	1	1.0	4	3.9	*P = 0.012; **P = 0.016	0	0.0	0	0.0	5	4.9	1	1.0		4	3.9	0	0.0	5	4.9	
		
	(250)	F	122	8	6.6	25	20.5		8	6.6	0	0.0	41	33.6	6	4.9		3	2.5	4	3.3	13	10.7	

V	0	M	117	2	1.7	1	0.9		0	0.0	5	4.3	8	6.8	0	0.0		1	0.9	1	0.9	2	1.7	
	
	(0)	F	102	7	6.9	9	8.8		2	2.0	9	8.8	27	26.5	4	3.9		1	1.0	7	6.9	12	11.8	


^a^ Percentages refer to the number of animals at start. HLRN data unpublished. **New statistical analyses (in bold):** * One-tailed Fisher’s exact test ; ** Two-tailed Fisher’s exact test. **# Cochran–Armitage test for trend**.

The following statistically significant increased trend was observed among treated groups: leukemias (all types) (MM: P = 0.0492 Cochran-Armitage trend test).

The following statistically significant increased incidences were observed in treated groups: animals bearing HLRN (MM: 16,000 ppm, P = 0.040 one-tailed Fisher’s Exact test), leukemias (all types) (MM: 32,000 ppm, P = 0.007 one-tailed Fisher’s Exact test, P = 0.009 two-tailed Fisher’s Exact test; 16,000 ppm, P = 0.001 one-tailed Fisher’s Exact test, P = 0.001 two-tailed Fisher’s Exact test; 8,000 ppm, P = 0.001 one-tailed Fisher’s Exact test, P = 0.001 two-tailed Fisher’s Exact test; 2,000 ppm, P = 0.018 one-tailed Fisher’s Exact test, P = 0.026 two-tailed Fisher’s Exact test; FF: 2,000 ppm, P = 0.007 one-tailed Fisher’s Exact test, P = 0.014 two-tailed Fisher’s Exact test), lymphoblastic leukemia (MM: 32,000 ppm, P = 0.021 one-tailed Fisher’s Exact test, P = 0.021 two-tailed Fisher’s Exact test; 16,000 ppm, P = 0.054 one-tailed Fisher’s Exact test, P = 0.054 two-tailed Fisher’s Exact test; 8,000 ppm, P = 0.001 one-tailed Fisher’s Exact test, P = 0.001 two-tailed Fisher’s Exact test; FF: 2,000 ppm, P = 0.012 one-tailed Fisher’s Exact test, P = 0.016 two-tailed Fisher’s Exact test), monocytic leukemia (MM: 16,000 ppm, P = 0.043 one-tailed Fisher’s Exact test, P = 0.043 two-tailed Fisher’s Exact test) and total myeloid tumours (MM: 16,000 ppm, P = 0.024 one-tailed Fisher’s Exact test, P = 0.024 two-tailed Fisher’s Exact test).

## Discussion

In the post-natal study on SD rats (BT6008), results are consistent with those already published, confirming the statistically significant increase of animals bearing HLRNs at doses of APM equal or higher than 400 ppm in female rats and a statistically significant increased trend in both male and female rats. Moreover, total lymphoid tumours were significantly increased in male and female rats at the highest dose of APM (100,000 ppm) and a statistically significant increased trend was observed in males. Total myeloid tumours were significantly increased in female rats at doses of APM equal or higher than 400 ppm and a statistically significant increased trend was observed in females. Statistically significant increased tumour incidences or trends were observed for the following subtypes: immunoblastic lymphoma (MM, FF), monocytic leukemia (FF), myeloid leukemia (FF) and histiocytic sarcoma (FF). Statistically significant increased tumour incidences or trends were observed in lymphomas (all types) (MM, FF) and leukemias (all types) (FF).

In the pre-natal study on SD rats (BT6009), results are consistent with those already published, confirming statistically significant increases of animals bearing HLRNs, lymphomas (all types) and leukemias (all types) in female rats treated with the highest dose of APM (2,000 ppm) and a statistically significant increased trend in female rats. Total lymphoid tumours were significantly increased in female rats at the highest dose of APM (2,000 ppm) and a statistically significant increased trend was observed. A statistically significant increased trend in total myeloid tumours was observed in female rats. Statistically significant increased tumour incidences or trends were observed for myeloid leukemia (FF).

In the pre-natal study on Swiss mice (BT6010), a statistically significant increase of animals bearing HLRNs at the dose of 16,000 ppm was observed in male mice. A statistically significant increase of leukemias (all types) in all groups treated with APM and a statistically significant increased trend was observed in males. In female mice, a statistically significant increase of leukemias (all types) was observed at 2,000 ppm. Total myeloid tumours were significantly increased in males at the dose of 16,000 ppm. Statistically significant increased tumour incidences or trends were observed for monocytic leukemia (MM) and lymphoblastic leukemia (MM, FF).

## Conclusion

Our analyses, performed in line with international recommended guidelines for statistics and pathology, confirm and reinforce our previous findings of statistically significant increases of HLRNs in rodents exposed to APM. Our analyses also confirmed that grouping HLRNs together tends to underestimate specific responses of different neoplasia types that become clearer when examined by lineage (myeloid vs lymphoid) or individual type. The presence of different highly significant results and dose-related increased trends in multiple studies highlight the biological relevance of our findings.
